# Can rheumatologists diagnose and manage Giant Cell Arteritis better than non-rheumatologists? The Maltese Experience

**DOI:** 10.31138/mjr.28.3.147

**Published:** 2017-09-29

**Authors:** Erika Cefai, Marica Galea, Rebecca Galea, Andrew A. Borg, Cecilia Mercieca

**Affiliations:** Department of Rheumatology, Mater Dei Hospital, Msida, Malta

**Keywords:** Giant cell arteritis, diagnosis, rheumatologists, incidence

## Abstract

**OBJECTIVES:**

Giant Cell Arteritis (GCA) remains a challenge both in terms of diagnosis and management as patients may present to several different specialists. The objectives were to determine incidence of biopsy-proven GCA in Malta and to compare the management between rheumatologists and non-rheumatologists.

**METHODS:**

This was a retrospective observational population study of patients with suspected GCA who underwent a temporal artery biopsy (TAB) between 2012 and 2015. Data collected consisted of demographics, presenting symptoms, TAB histology reports, treatment and outcome. The British Society for Rheumatology (BSR) 2010 guidelines were used as standard of care.

**RESULTS:**

136 patients underwent a TAB for suspected GCA of which 26 were positive. The incidence of biopsy-proven GCA in Malta was 3.82 per 100,000 patient years in the over 50 population. There were 63 patients who were treated as GCA. Only 43.3% of confirmed cases had rheumatology input. TABs requested by rheumatologists were twice more likely to be positive compared to requests by non-rheumatologists (30.5% vs. 14.1%).The majority of patients were started on a Prednisolone dose between 40–60mg. Rheumatologists maintained patients on high doses for at least 1 month in 54% of cases as opposed to 20% under non-rheumatologists. Monitoring was more regular for cases followed up by rheumatologists (40% vs. 21%).

**CONCLUSIONS:**

Malta has a low incidence of biopsy proven GCA. Although rheumatologists are more likely to adhere to the recommended guidelines, improvement is needed. Rheumatologists should take the lead to minimise variation and optimise management of GCA.

## INTRODUCTION

GCA is the most common vasculitis diagnosed in adults over the age of 50 years, affecting the large- and medium-sized arteries and 70% of sufferers are female.^1^ It predominantly affects the extra-cranial branches arising from the carotid artery but a proportion of patients have aortic involvement.^2^ The highest incidence rates of GCA are reported in Scandinavian countries where incidence is >17 per 100,000 person-years in the population age ≥50 years.^3^ In Mediterranean countries, the incidence is lower, typically <12 per 100,000 person-years in the same age group. Very low annual incidence rates are reported in Turkey and Japan, at 1.13 per 100,000 person-years and 1.47 per 100,000 person-years, respectively.^3,4^ Biopsy proven GCA is associated with higher short-term mortality especially in patients diagnosed under the age of 70.^5^ Amongst the potential complications of GCA, the one that is of greatest concern is permanent blindness.^6^ The incidence of permanent sight loss is 4% and anterior ischaemic optic neuropathy is 8%.^7^ The most common symptoms at presentation include new onset or temporal headache, scalp tenderness and jaw claudication accompanied by an inflammatory response.

The diagnosis and management of GCA remains a challenge for a variety of reasons. There are many conditions that mimic GCA such as sepsis, malignancy and other forms of vasculitis. Secondly, patients present acutely to a wide variety of specialists which may result in significant variations in clinical care and treatment delay. Lastly, treatment needs to be taken for at least a couple of years which makes adherence to medication very important. The complications of GCA can be potentially very devastating but can be reduced by means of timely diagnosis and appropriate treatment. To date there is a dearth of literature looking at the proportion of GCA patients managed by rheumatologists and non-rheumatologists and whether there are any differences in clinical practice. This is important in order to target interventions and improve quality of care.

This was a retrospective observational 4-year population study with the objectives to determine the incidence of GCA in the Maltese population and to assess whether rheumatologists are better able to diagnose and manage this condition compared to non-rheumatologists.

## MATERIALS AND METHODS

All Maltese patients who underwent a TAB for suspected GCA between the years 2012 and 2015 were included in the study. The data collected was obtained by reviewing the case notes and included demographics, co-morbidities, presenting and caring specialities, symptoms at presentation, investigations, treatment and outcome. A biopsy specimen was considered positive if there was a mononuclear cell infiltrate or granulomatous inflammation, with mononucleated giant cells. Mortality at 1 year from diagnosis was assessed. The data was then analysed and compared so as to identify management differences between rheumatologists and non-rheumatology specialities. The BSR guidelines for management of GCA published in 2010 were used as the standard of care.^8^ These guidelines were chosen as they include the most recent evidenced-based recommendations for the diagnosis and management of GCA.

## RESULTS

**Figure 1:**
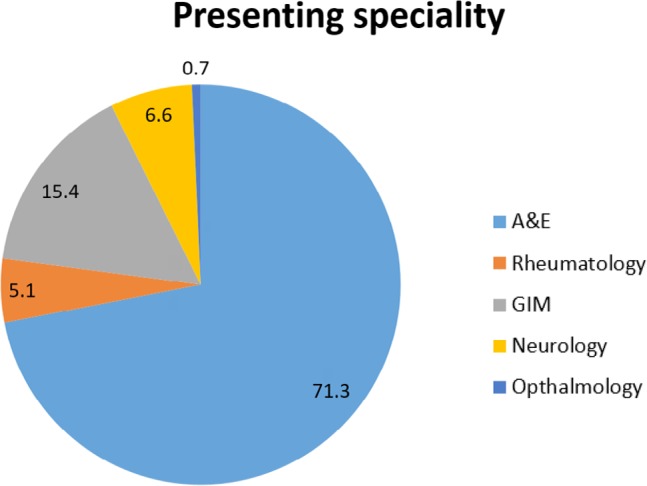
The proportion (%) of patients with suspected GCA presenting to the various specialities

**Figure 2:**
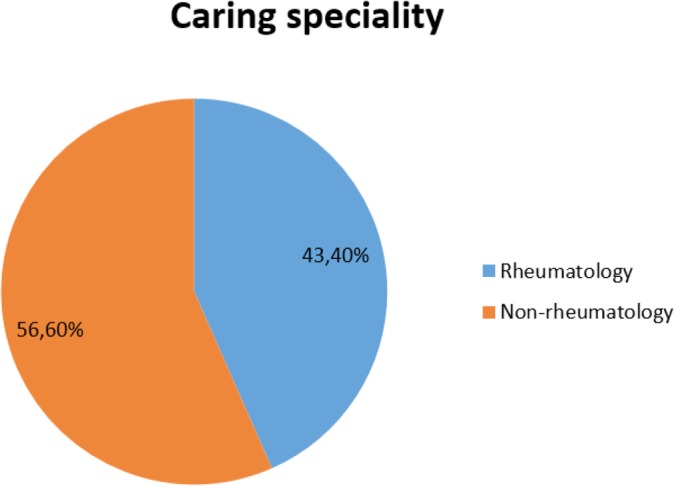
Proportion (%) of patients diagnosed with GCA (n=63) managed by rheumatologists and non-rheumatologists

There were 136 patients (90 females) who underwent a TAB. The mean age was 72.4 (SD 10.2) years. The majority of patients with suspected GCA presented to the emergency department (71.3%) whilst the remaining patients presented directly to other specialities including general internal medicine (15.4%), neurology (6.6%), rheumatology (5.1%) and ophthalmology (0.7%). At presentation, 43.3% of patients with confirmed GCA were referred to a rheumatologist whilst the rest remained under the care of the other specialists without rheumatology input*.* (**Figures 1** and **2**)

**Figure 3:**
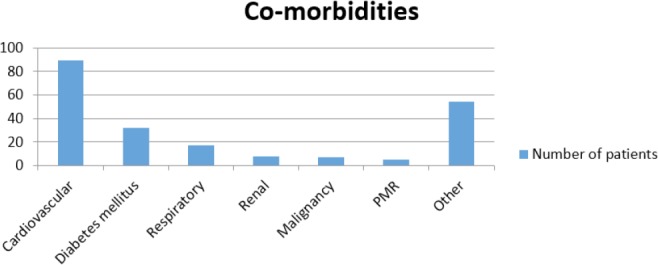
Presence of co-morbidities for suspected GCA cases

The most common co-morbidities, in decreasing frequency, were cardiovascular, diabetes mellitus, chronic kidney disease, malignancy, respiratory disorders and Polymyalgia Rheumatica (PMR) either alone or in combination. (**Figure 3**)

There were 26 positive biopsies (19.1%). Out of the 59 TABs requested by the rheumatologists 30.5% were positive, whilst 14.1% of TABs requested by non-rheumatologists were positive. There were 37 patients who had negative biopsies but were treated for GCA on clinical grounds. The main differential diagnoses for suspected GCA cases included sepsis (n=7), PMR (n=5), pyrexia of unknown origin (n=4) and cluster headaches (n=3).

At 1 year post-diagnosis, two patients had died. The causes of death were pulmonary embolism and uterine malignancy. Both patients had multiple comorbidities including cardiovascular disease, diabetes and chronic kidney disease.

**Figure 4:**
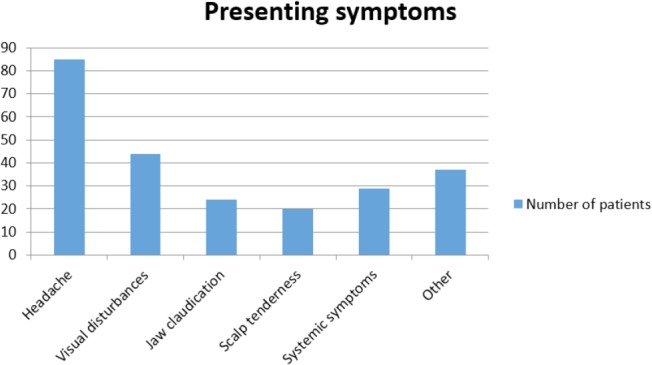
The frequency of the various presenting symptoms of patients with suspected GCA.

The mean duration of symptoms from onset to presentation for patients diagnosed with GCA was 19.2 days (SD 15.10). The most common symptoms at presentation that raised suspicion of GCA were headaches (n=85), visual disturbances (n=44), jaw claudication (n=24), scalp pain (n=20) and systemic symptoms (n=27) either alone or in combination. Systemic symptoms included fever, weight loss, night sweats and low appetite. Amongst the visual disturbances reported there were double vision, reduced visual acuity and amaurosis fugax. Two patients presented with visual loss. The first case had complete unilateral visual loss whilst the second had an inferior visual field defect. (**Figure 4**)

All patients had an Erythrocyte Sedimentation Rate (ESR) and C-reactive protein (CRP) recorded at presentation. The mean ESR was 77.1mm/hr (SD 35.8) and CRP was 64.6 mg/L (SD 10.2) respectively. Mean CRP value in the biopsy-proven GCA cohort was 101mg/L vs. 47.5mg/L in the biopsy-negative GCA cohort. Out of 59 cases treated as GCA, 7 had a normal ESR and CRP at baseline, 13 had an elevated ESR with a normal CRP and the remaining 39 cases had both ESR and CRP elevated.

Only, 5.8% of patients presented directly to a rheumatologist or ophthalmologist while less than half (43.4%) were eventually referred on to a rheumatologist. High-dose glucocorticoids were initiated in 36% of suspected cases (n=49) prior to undergoing TAB and of these 49% were given by rheumatologists.

A fifth of TAB were positive for GCA and the majority of these were requested by rheumatologists (69.2%). The mean length of all the biopsy specimens was 8.9mm (range 2–25mm).Over half of TAB (56%) were less than 1 cm in length: 67.3% of these were negative (n=66/110) and 44% were positive (n=11/26). Only 1 biopsy was longer than 2cm (25mm) and it was reported negative but the patient was still treated on clinical grounds.

A third of patients with suspected GCA (36%) received glucocorticoid therapy before undergoing a TAB. Eight patients received intravenous methylprednisolone, in most cases this was given by rheumatologists (75%).

A prednisolone starting dose between 40–60mg was given to 86% of cases managed by rheumatologists and 82% of cases treated by non- rheumatologists. Three patients with GCA treated all by non-rheumatologists received doses exceeding the recommended 60mg.

A wide variation of tailoring regimes were noted and documentation was poor particularly by non-rheumatologists. Rheumatologists treated 54% of patients with a prednisolone dose of 40mg or greater for at least one month, 36% received lower doses and in 10% it was unclear. (**Table 1**)

**Table 1:** Glucocorticoid treatment regimens prescribed for GCA cases.

	Rheumatology (n= 35)	Non rheumatology (n=28)
Prednisolone 40–60mg +/− IV methylprednisolone	86%	82%
Prednisolone less than 40mg	14%	7%
Prednisolone more than 60mg	0	11%

Fluoro-deoxyglucose Positron Emission Tomography (FDG-PET) was requested for 2 patients who had presented with pyrexia of unknown origin and both were under rheumatology care. Both cases had diagnostic PET scans.

Only 19 patients diagnosed with GCA had regular monitoring of their inflammatory markers and were followed up at least every 4 months. The mean time between visits was 11.7 (SD2.8) weeks. Regular monitoring occurred in 40% under rheumatology care against 21% managed by non-rheumatologists. CRP level at 1 year post diagnosis, was checked for all GCA cases and was normal in 45.7% and 14.3% of rheumatology and non-rheumatology GCA cases respectively.

## DISCUSSION

The incidence of biopsy proven temporal arteritis in Malta was 3.82 per 100,000 person years aged over 50 years.^9^ This is comparable to incidence rates reported in other Mediterranean countries.^10^

It is well known that GCA patients present to a wide variety of specialists. To our knowledge this is the first study looking at the management of GCA by different specialists. This enables a more realistic assessment of what happens in daily practice and identifies opportunities of how care could be improved. In this study, a high proportion of patients were referred to the emergency department where they were assessed by emergency physicians. This suggests that there is significant awareness amongst general practitioners (GP) about the importance to refer patients with suspected GCA for urgent assessment and treatment. Nonetheless, there was a significant time lag from onset of symptoms to presentation. This could be due to patients’ factors as well as GP factors, such as low index of suspicion which is understandable, given the variable and sometimes insidious presentation of GCA. The vast majority of patients with suspected GCA seen at the emergency department were admitted under medical care or in case of visual loss to ophthalmology. Surprisingly, rheumatologists were involved less than half of suspected GCA cases. Although the rheumatology department offers a daily on call service that is run by a team of 5 consultants there still isn’t a direct referral system/established care pathway from GP to rheumatology during out of hours. The latter could partially explain why there was a substantial number of cases that remained without rheumatology input. Alternatively, GCA may be seen as a condition that can be adequately managed by non-rheumatologists. These results are in contrast with BSR guidance which recommend that all patients with suspected GCA should be evaluated by a specialist, i.e., a rheumatologist/ophthalmologist prior to performing a TAB. The diagnosis of GCA is often challenging, even in the hands of an experienced rheumatologist. A diagnosis of GCA implies long term treatment with glucocorticoids which is not a decision to be taken lightly. In fact GCA is one of the commonest conditions requiring long term high to moderate doses followed by gradual tapering to low doses. TAB still remains the gold standard investigation. Prior to performing a TAB and initiating glucocorticoid treatment it is important to be clear about the probability of GCA as a negative biopsy does not exclude the diagnosis. Additionally, treatment with glucocorticoids until the biopsy result is available (which usually takes about a week), may cloud the picture even further. A number of clinical predictors have been identified including headache, jaw claudication, ESR and thrombocyte levels.^11^ These can be of use in differentiating patients with low suspicion of GCA from those with medium/high suspicion. Patients with low probability should not have a TAB. We recognise that some cases treated as GCA who were not referred for a TAB or not coded as GCA were not included in our study. Unfortunately, there was no way we could capture these patients. There was a clear difference in the number of positive TAB requested by rheumatologists and non-rheumatologists suggesting, that non-rheumatologists may have a low threshold for requesting TAB or that rheumatologists maybe better at assessing and diagnosing GCA. By involving a rheumatologist prior to performing a TAB a number of unnecessary TABs may be reduced.

Biopsy length seems to be an important factor which may be overlooked. In order to maximise the diagnostic yield a TAB should be more than 1cm in length, preferably up to 2cm due to presence of skip lesions.^8,12^ The mean biopsy length, in this study, was below the minimum recommended length recommended by BSR and EULAR which could have affected the overall results.^8,12^ In our study, TABs were performed on the emergency surgical lists by surgical trainees. While on one hand this enabled timely biopsy, this may have led to the high number of biopsies below the recommended length. Having a clear protocol and surgical engagement may help ensure that an adequate length is obtained.

The most common documented co morbidities were cardiovascular complications and diabetes mellitus. This has important implications both prognostically and as regards treatment regimens with glucocorticoids. Presence of comorbidities maybe one of the reasons why clinicians might prescribe low glucocorticoid doses and/ or opt for fast tailoring regimens. Labarca et al. reported that female gender and baseline hypertension and/ or diabetes can portend to a higher frequency of relapse in biopsy-proven GCA.^13^ In clinical practice one has to balance the risk of adverse effects of glucocorticoids with the risk of under treatment or relapse which may eventually result in higher doses and prolonged course of glucocorticoids. It has been shown, that up to 50% flare during glucocorticoid tapering requiring escalation and a more prolonged treatment course.^7^ Glucocorticoid-sparing agents such as methotrexate may offer an alternative therapeutic option for patients who cannot tolerate or are high-risk for taking high-dose glucocorticoid therapy as they may allow faster tapering and lower cumulative doses. However, the evidence supporting use of Methotrexate is not strong. There is some evidence supporting use of Mycophenolate Mofetil for treatment of large vessel vasculitis and anti-interleukin 6 receptor therapy.^14^ Again, more studies are required to support use of these newer therapies. Interleukin 6 (IL-6) production has been found to be enhanced in TAB samples besides correlating well with disease activity. IL-6 blockade may represent a form of novel rescue therapy for patients with relapsing or refractory GCA.^15^

While it is clear that high doses of glucocorticoids are required to suppress GCA and maintain remission, the lack of robust evidence supporting specific glucocorticoid doses and tailoring regimens may contribute to the variation experienced in clinical practice. Lack of clinical experience about the varying doses and modes of action of glucocorticoids and misplaced concern about adverse effects could be another barrier to appropriate use of glucocorticoids. BSR guidance recommends keeping the patient on a dose of 40–60mg prednisolone until there is clinical resolution of symptoms and improvement in laboratory abnormalities. While both rheumatologists and non-rheumatologists treated the vast majority of patients with the proper recommended high glucocorticoid dose, non-rheumatologists were less compliant with the recommended tailoring regimens and monitoring. Non-rheumatologists seemed to give less importance to follow up visits and monitoring of inflammatory markers. Regular monitoring is key to ensure compliance, response to treatment, identify adverse effects or new complications. CRP remains a more sensitive diagnostic marker for GCA and it should be regularly monitored together with clinical assessments.^16^ ESR and CRP are two important investigations that are required both at baseline and during follow up of patients with GCA. Interestingly, it is recognised that there are patients with biopsy- proven GCA who have a normal inflammatory markers. Kermani TA et al. reports a frequency of this occurring of 4–14% which is similar to the 11.9% obtained in this study.^17^

The role of PET-CT in the diagnosis of GCA is becoming increasingly important though it is still not recommended to perform such an investigation for every patient with suspected GCA. In this study, rheumatologists requested it in setting of an investigation of a PUO where the diagnosis was still unclear and the patients had not yet received glucocorticoid therapy.

This was the first study looking at the incidence of biopsy-proven GCA in Malta. The incidence may have been underestimated as there could have been cases which were treated purely on clinical grounds without ever having a biopsy. Additionally, since the quality of certain TABs was sub-optimal the number of positive biopsies could have been underestimated. Given the retrospective nature one of the main limiting factors was lack of documentation. On the other hand various aspects pertaining to diagnosis and management of GCA were assessed in detail and compared between rheumatologists and non-rheumatologists giving a more realistic picture of what happens in clinical practice. To our knowledge, this has not been assessed in other studies.

**Figure 5:**
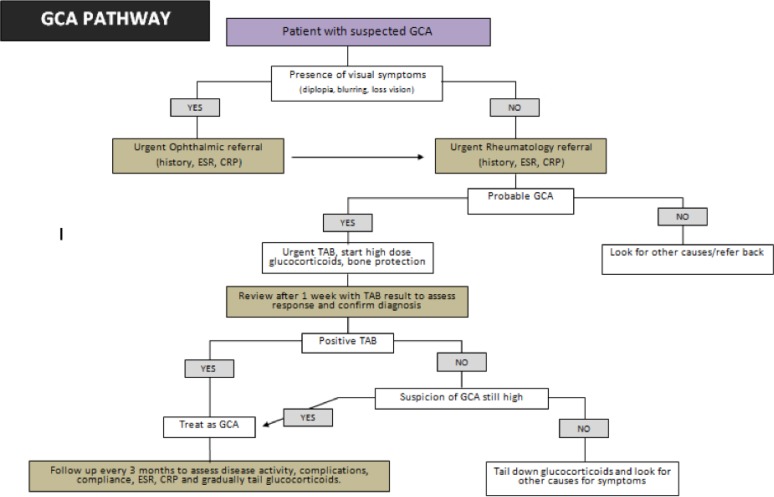
Proposed GCA pathway

Developing an integrated care pathway involving all the relevant stakeholders might help improve the standard of care. In our opinion, patients who are suspected of having GCA based on the history, examination findings and investigations should be urgently assessed by a rheumatologist who will then take the lead and decide regarding the need to perform a TAB and initiate glucocorticoid treatment. We propose the following pathway (**Figure 5**). The next phase of the study will be to obtain long-term data for GCA cases treated by rheumatologists and non-rheumatologists so as to see if the differences highlighted above have an impact in terms of morbidity and mortality. This will involve looking into aspects such as compliance and adherence to treatment, adverse effects, hospitalisations and deaths.

## CONCLUSION

Rheumatologists were overall better able to assess, diagnose, treat and follow-up patients with GCA when compared to non-rheumatologists. However, there are many aspects that need improvement particularly monitoring and treatment regimens. Non rheumatologists, appear to have a low threshold to request TABs. In order to ensure timely and appropriate management rheumatologists should take the lead and set up interspecialist pathways based on international guidelines that work in the local context. Such pathways should include guidance about access, diagnosis, investigations and treatment which should be disseminated to other colleagues in both primary and secondary care.

## ABBREVIATIONS

BSR: British Society of Rheumatology
CRP: C reactive protein
ESR: Erythrocyte Sedimentation Rate
EULAR: European League against Rheumatism
FDG-PET: Fluorodeoxyglucose Positron Emission Tomography
GCA: Giant cell arteritis
GP: general practitioner
IL-6: Interleukin 6
PMR: Polymyalgia Rheumatica
TAB: temporal artery biopsy

## References

[1] Borchers A T Gershwin M E. Giant cell arteritis: a review of classification, pathophysiology, geoepidemiology and treatment. Autoimmune Rev 2012;11:A544–54. 10.1016/j.autrev.2012.01.003 22285588

[2] Baslund B Mortality in patients with giant cell arteritis. Rheumatology 2015;54:139–43. 25122725 10.1093/rheumatology/keu303

[3] Jakobsson K Body mass index and the risk of giant cell arteritis- results from a prospective study. Rheumatology 2015;54:433–40. 25193806 10.1093/rheumatology/keu331 PMC4425830

[4] Petri H Incidence of Giant Cell Arteritis and characteristics of patients: Data-driven analysis of comorbidities. Arthritis Care Res 2015;67:390–5. 10.1002/acr.22429 25132663

[5] Mohammad J Incidence and mortality rates of biopsy proven GCA in southern Sweden. Ann Rheumatic Dis 2015; 74:993–7. 10.1136/annrheumdis-2013-204652 24442881

[6] Mollan S P Increase in admissions related to giant cell arteritis and polymyalgia rheumatic in the UK, 2002–13 without a decrease in associated sight loss: potential implications to service provision. Rheumatology 2015;54:375–7. 25413943 10.1093/rheumatology/keu433

[7] Singh Abha J Visual manifestations in GCA: Trend over five decades in a population-based cohort. J. Rheumatology 2015 2; 42:309–15. 10.3899/jrheum.140188 PMC4367485 25512481

[8] Baskhar D BSR and BHPR guidelines for management of Giant Cell Arteritis; Rheumatology 2010;49:1594–7. 20371504 10.1093/rheumatology/keq039a

[9] Demographic Review 2013- Valletta: National Statistics Office, 2015.

[10] Mohammad A J Ann Rheum Dis. 2015;74:993–7. 24442881 10.1136/annrheumdis-2013-204652

[11] Grossman C Baseline clinical predictors of an ultimate giant cell arteritis diagnosis in patients referred to temporal artery biopsy. Clin Rheumatol 2016;35:1817. 26925851 10.1007/s10067-016-3221-1

[12] Mukhtyar C EULAR recommendations for the management of large vessel vasculitis. Ann Rheum Dis 2009; 68:318–23. 18413441 10.1136/ard.2008.088351

[13] Labarca C Predictors of relapse and treatment outcomes in a biopsy-proven giant cell arteritis: a retrospective cohort study. Rheumatology 2016;55:347–56. 26385368 10.1093/rheumatology/kev348 PMC4939727

[14] Smith R Is Mycophenolate Mofetil effective in the treatment of large vessel vasculitis? Ann Rheum Dis 2015;74:525.

[15] Unizony S Tocilizumab for treatment of large-vessel vasculitis (GCA, Takayasu’s arteritis) and Polymyalgia Rheumatica. Arthritis Care Res 2012;64:1720–9. 10.1002/acr.21750 22674883

[16] Jakobbson K The effect of clinical features and glucocorticoids on biopsy findings in giant cell arteritis. BMC Musculoskeletal Disorders 2016;8 24. 10.1186/s12891-016-1225-2 PMC4997683 27558589

[17] Kermani T Schmidt J Crowson C S Ytterberg S R Hunder G G Matteson E Utility of Erythrocyte Sedimentation Rate and C-reactive protein for the Diagnosis of Giant Cell Arteritis. Semin Arthritis Rheum 2012;41:866–71. 22119103 10.1016/j.semarthrit.2011.10.005 PMC3307891

